# ‘As though Miles of Ocean did not Separate us’: Print and the Construction of a Transatlantic Free Love Community at the Fin de Siècle

**DOI:** 10.1093/jvcult/vcz054

**Published:** 2019-11-29

**Authors:** Sarah L Jones

**Affiliations:** Department of History, University of Exeter

**Keywords:** print culture, free love, sex radicalism, periodicals, social reform, transatlantic

## Abstract

This article argues that British and American free lovers – radical sexual reformers committed to the cause of ‘sexual freedom’ – came together through print to build a transatlantic community at the fin de siècle. Challenging existing narratives that characterize free love as isolated or incoherent, it argues that through print free lovers from Britain and America were able to forge links with each other, and to construct an important, coherent collective identity that transcended national boundaries. In doing so it makes two major interventions. First, it provides unique new insights into the history of free love in both the British and American contexts, placing a new focus on often overlooked transnational connections and exchanges that helped to shape late nineteenth-century free love campaigns. Second, it encourages historians to rethink the ways we look for and make sense of cohesive international reform communities more broadly in this period. By exploring how a small, radical group like the free lovers were able to cohere through processes of contestation and negotiation played out entirely in print, this article will show that, where necessary, print was enough for transatlantic reformers to construct common identities and negotiate coherent reform ideas. As such, it argues that historians of fin-de-siècle social reform should look again at the print culture of other contemporary reformers otherwise labelled divided, isolated, or marginalized to look for threads of cohesion, cooperation, and compromise.

In the summer of 1898, the secretary of the British free love organization The Legitimation League was charged with publishing obscene articles on free love and radical sexual reform in *The Adult –* the League’s monthly paper of which he served as editor.^1^ George Bedborough’s American friends were quick to object to the charges against him: Moses Harman, the editor of prominent American free love periodical *Lucifer, the Light-Bearer*, spearheaded a campaign to fund his defence, calling on his readers to donate to what he saw as a common, transnational cause. Urging American supporters to support their ‘comrade’ Bedborough, Harman emphasized that they were part of a broader, transatlantic free love network: ‘Every lover of freedom in America should feel an interest in the Bedborough case’, he declared, ‘for the cause of freedom is one and is not divided by geographical lines’.^2^ Harman’s support for Bedborough in this time of crisis was not an isolated incident and was part of sustained attempts to forge a relationship between sex radical reformers on both sides of the Atlantic at the fin de siècle. These reformers (who will be referred to as either sex radicals or free lovers in this article)^3^ frequently asserted in their writings that they were part of the same cohesive community; ‘an association international in character’ campaigning together for a common, if somewhat ambiguous, cause – ‘sexual freedom and the essential purity of the body and its functions’.^4^

The relationship was not necessarily an easy one to build and maintain, however. British and American free love movements were, of course, influenced by very different national contexts at this time; their separate agitations for radical sexual reform were certainly shaped by different sets of traditions and motivations tied to the specific social, cultural, and political landscapes of late nineteenth-century Britain and America. Importantly, Anglo-American free lovers also lacked what Lucy Delap has called the ‘glue’ that held together and shaped many other international reform communities around this time – the lecture tours, personal connections and friendships, and international conferences that allowed opportunities for face-to-face discussion.^5^ Consistently financially precarious and lacking the wider support enjoyed by bigger, better-known contemporary reform streams like suffrage and abolition, free lovers were for the most part unable to facilitate their transatlantic connections through these more expensive, direct means that would have tested their already tight budgets. Indeed, records suggest that British and American sex radicals only met in person twice over the entire course of their near decade-long association. They were therefore compelled to find alternative ways to sustain an international relationship: capitalizing on technological innovations that made transatlantic communication increasingly easy, as well as a late nineteenth-century boom in periodical production, they relied entirely on print.

But how, exactly, did such a diverse, widely dispersed, and potentially disparate group of reformers manage to build an international community through print alone? To explain, this article will focus on the relationship between two prominent free love journals active in the closing years of the nineteenth century: Bedborough’s *The Adult*, the journal of the Legitimation League published from London from 1897, and the *Lucifer, the Light-Bearer*, published by Harman from Topeka, then Chicago, from 1883. My analysis of these journals and associated publications will show that print allowed free lovers to construct an international community in two main ways. To begin, building upon work by scholars like Lucy Delap and John Fagg into the construction of periodical communities in more prominent contemporary reform networks, it will demonstrate how exchanges, collaborations, and conversations characteristic of contemporary print culture facilitated practical connections between seemingly very separate and different radical groups.^6^ Challenging narratives of contemporary sex radicalism that focus on a single national context, it will show that, through print, free lovers were able to establish a rich and lively transatlantic reform community. Following this, it will show that print provided these far-flung free lovers with an important rhetorical space in which they could negotiate and compromise on their radical views about sex; in the pages of their journals a diverse range of free love advocates were able to enter shared conversations about the problems of marriage, and to cultivate a common vocabulary with which to discuss freedom in sexual relationships. It will chart the construction of a common identity by looking at the specific ways in which, lacking other spaces to negotiate their ideas, British and American free lovers were able to construct a shared vision for radical sexual reform entirely through print. In essence it will show that despite difference and distance, free lovers on both sides of the Atlantic were not irreparably separate. Instead, they should be understood as a cohesive reform community campaigning together for radical changes to sex and marriage at this time.

In doing so, this article makes two major interventions. First, it provides unique new insights into the history of free love in both the British and American contexts. The existing scholarship on Britain, for example, has often focused on prominent free unions occurring on the margins of broader social reform movements, such as those for suffrage or socialism.^7^ Studying free unions as an extreme form of protest hovering at the most radical edges of other movements has meant that free love in Britain tends to exist in the historical imagination largely as the protest action of a select, zealous few. As such, historians have generally approached it as something scattered and incoherent – a peculiar, unpopular, or hard-line form of rebellion regularly confined to the very margins of historical analysis. Even narratives that take a broader approach, such as Ginger Frost’s rich analysis of unmarried cohabitation in the nineteenth century, focus heavily on the disjointed nature of contemporary sex radicalism, and the deep divisions and contradictions between those advocating for sexual reform.^8^ Shifting the focus towards print, however, can tell a new story; challenging one-dimensional characterizations of free love as something unquestionably incoherent it demonstrates for the first time that, in Britain, free love was part of a cohesive international reform network with a collective identity and distinct set of reform ideas of their own. While the historiography of American free love has generally paid more attention to the depth and complexity of radical sexual reform movements, as well as the important role of print, it has nevertheless remained focused on a single national context.^9^ Limited in scope to looking only within American borders, much of this scholarship gives the impression that the campaigns for free love and sexual liberty occurring in the last decades of the nineteenth century were somehow peculiarly American – a product of only American advocates, and isolated specifically to the social, cultural, and political climate of the United States. Charting the creation of a transatlantic print community ties the history of free love to broader histories of transatlantic reform by scholars such as Daniel Rodgers and Leslie Butler, placing a new and important focus on often overlooked transnational connections and exchanges that helped to shape progressive-era free love campaigns.^10^

But a focus on the particular role of print in building communities out of such a diverse group of reformers does more than demonstrate the transnational nature of free love. I argue that concentrating on print, and especially periodicals – a medium Margaret Beetham states helped people ‘find common ground, [and] recognise each other across differences of space and time’ – can encourage us to rethink the ways we look for and make sense of cohesive international reform communities more broadly in this period.^11^ Many accounts of larger and better-funded contemporary reform movements emphasize the importance of looking at personal connections and international travel in order to understand deepening global links.^12^ Even Lucy Delap’s assertions of the importance of periodical networks to early twentieth-century feminism situates these networks within a broader context of international friendships and visitation.^13^ By exploring how a small, radical group like the free lovers were able to cohere through processes of contestation and negotiation played out entirely in print, this article will expand on important work being done into nineteenth-century print culture to show that, where necessary, print *was* enough for transatlantic reformers to construct common identities and negotiate coherent reform ideas at this time. As such, it argues that historians of fin-de-siècle social reform should look again at the print culture of other contemporary reformers otherwise labelled divided, isolated, or marginalized to look for threads of cohesion, cooperation, and compromise.

## 1. FORGING A RELATIONSHIP THROUGH PRINT

Factors such as technological innovations, falling production costs, and increasing literacy rates ensured that the late nineteenth century was a ‘watershed moment’ in the rise of print production.^14^ Within this context, free lovers in both Britain and America produced a huge amount of literature on topics around sex and relationships to support their calls for radical sexual reform. In addition to the creation of a wealth of texts like advice books and novels, these free lovers also published their own periodicals – most notably Harman’s *Lucifer, the Light-Bearer*, and the Legitimation League’s *Adult*.^15^ These periodicals acted as important arenas for discussion for this diverse group: as Laurel Brake and Julie F. Codell have shown, nineteenth-century periodicals often helped to facilitate ‘encounters’ between diverse contributors with individual and contrasting views.^16^ For free lovers unable to facilitate such ‘encounters’ elsewhere, this meant that they acted as an especially important shared space for reformers from different backgrounds, with different motivations, working in different contexts, to connect with each other and negotiate their complex views about marital and sexual reform. In short, they ensured that a community of readers, writers, and correspondents on both sides of the Atlantic were able to participate in a shared conversation about the benefits of radical sexual reform and what it meant to be sexually free. Though existing research into free love has often overlooked such exchanges in favour of accounts of a single national context, I argue that at the end of the nineteenth century free lovers were able to establish a transatlantic community through connections forged solely in print.

As scholars such as Ann Ardis and Patrick Collier have shown, the free lovers were certainly not alone amongst their contemporaries in using print in this way; other important transatlantic reform communities from the period such as those dedicated to temperance, suffrage, and abolition also looked to print to support and shape their campaigns, and to draw sympathetic reformers in different national contexts together.^17^ Indeed, Amanda Claybaugh has argued that much contemporary social reform turned to print precisely for its ability to transcend national boundaries, and to allow reformist ideas to enter culture more broadly.^18^ However, print was even more important for small, radical, and diffuse groups like the free lovers. Both American and British groups struggled financially from the outset; heavy fines and periods of imprisonment imposed upon Harman and *Lucifer* by evangelical vice reformer Anthony Comstock strained already limited funds in America, for example, while *The Adult* was largely funded by the personal fortune of a single donor, who went bankrupt in 1899.^19^ Beyond their financial insecurity, it was also difficult for those who shared a commitment to radical sexual reform to publicly advocate free love in this period due to fears of being accused of licentiousness or ‘free lust’. As Joanne Passet has noted, many free lovers (particularly women) thus operated ‘undercover’ to protect their reputations, and were largely unwilling to participate in sex radical agitation in person.^20^ Therefore, while international travel, conferences, and lectures may have been productive, they were nevertheless out of the reach of many of the free lovers who needed to find cheaper, more sustainable, and more discreet ways to build connections. Following Passet’s study of grassroots feminism in the contemporary American context, I argue that print therefore played an especially vital role in constructing a community out of an otherwise potentially disparate, marginalized and widely dispersed group; helping to develop a sense of comradeship and connection between reformers that allowed free love to develop beyond borders.^21^ Interrogating free love print networks, then, offers a unique insight into the particular role of print in facilitating the construction of transatlantic reform communities at the fin de siècle.

The circulation of texts, the reprinting of content, and the sharing of authors played a particularly key role in overcoming differences between British and American free love circles. In the context of social, economic, and technological changes that ensured that print in the transatlantic arena was becoming an increasingly ‘entangled ecosystem’, such texts moved easily and comparatively cheaply over borders, allowing, as Bob Nicholson has argued, ideas and approaches from one country to enter the ‘cultural bloodstream’ of the other – a critical tool for diverse free lovers unable to cultivate connections in person and lacking opportunities to negotiate their ideas face to face.^22^ Sympathetic to many of the calls for sexual freedom circulating in the United States, for example, from its first issue in 1897 *The Adult* made highlights of American sex radical literature available to their readers by reprinting them in its pages. Though produced in a different national context, contributions by American authors were heavily featured in *The Adult*, such as work by editor Moses Harman and his daughter Lillian, alongside reproductions of articles by American free lovers such as the anarchist E. C. Walker and Tennessee Claflin, co-editor of pioneering free-love paper *Woodhull & Claflin’s Weekly*.^23^*Lucifer* operated in a mutually supportive way, publishing articles written by British free lovers on such topics as censorship, monogamy, and freethought. Indeed, the 28 May 1898 edition of *Lucifer* was labelled by its editor as ‘The Legitimation League number’ due to prevalence of British contributions, praised for their ‘close relation to *Lucifer’s* main line of work’.^24^

Furthermore, British and American free lovers also worked together to ensure the transatlantic reach of free love literature by acting as advertisers and distributors for each other’s work. By facilitating access to both British and American sex radical texts they made certain that the work of free lovers was not constrained by national boundaries, and that a geographically diverse range of sex radicals had access to a common pool of texts. In part this was as simple as acting as agents for one another: prominent *Lucifer* collaborator E. C. Walker became *The Adult*’s American distributor shortly after its launch, for instance, while the Legitimation League sold a large variety of American sex radical books, periodicals, and pamphlets from their offices in London. These networks of distribution certainly helped foster a sense of belonging, or at least of shared enterprise; William Duff, a member of the Legitimation League who acted as an agent for *Lucifer* in Glasgow, for example, told his American associates that he saw his agency as a way to ‘spread the truths we all have at heart, or perhaps I should say, in mind’.^25^ Such a network of texts, authors, and distributors therefore played an important role in creating a sense of cohesion – ensuring that, for all their differences, free lovers from both sides of the Atlantic were consuming the same literature, encountering the same ideas, engaging in the same debates, and developing a common vocabulary through which to discuss their ideas about sexual reform.

In addition to the exchange of ideas facilitated by the sharing of articles and key texts discussed above, periodicals, in particular, helped to facilitate dialogue between free lovers operating on both sides of the Atlantic. It was common for correspondents from both Britain and America to write to the journals in order to offer comments on each other’s work, to respond to questions or challenges raised, or to engage in transatlantic debates about various aspects of their reform work – in essence, allowing them to engage with each other as if they weren’t separated by thousands of miles. Through *Lucifer*, for example, British free lover John Badcock Jr, writing in London, was able to engage in a heated discussion with American correspondent William Gilmour about the protection of unmarried mothers,^26^ while the pages of *The Adult* allowed English free lover William Platt space to confront Chicago-based Lillian Harman over her comments on the payment of women for domestic labour.^27^ As well as allowing them to challenge and debate each other from afar, letters published in correspondence sections such as *The Adult’s* ‘Our Letter Box and *Lucifer*’s ‘Various Voices’ allowed radicals to pledge support for each other, or to praise each other’s reform efforts; to send, as *Adult* editor George Bedborough did in 1897, ‘hearty handshakes’ to their transatlantic friends ‘as though miles of ocean did not separate us’.^28^

The regular exchange of letters, reviews, and comment pieces facilitated by these journals is testament to what Brake and Codell call the ‘multi-vocal’ nature of contemporary periodicals; but in this instance they also demonstrate how dialogue facilitated by print played a crucial role in the construction of a sense of cohesion and shared endeavour.^29^ As Jesse Battan has shown, the extensive correspondence between sex radicals through periodicals was often instrumental in the formation of a ‘common community of belief and action’.^30^ While Battan’s work only considers this process in the context of American free love circles, letters also helped to create an Anglo-American free love community out of a group of reformers with few other opportunities to connect. Though these transatlantic connections have often been ignored in existing historical literature, readers in both Britain and America offered their support and encouragement, exchanged ideas and opinions, and actively participated in a dialogue about free love through their correspondence with journals. Regardless of the different national contexts they inhabited, the relationship between these free love periodicals was instrumental in helping to build a sense of community between readers that was based, as Battan has noted, ‘not on geographical proximity but instead on common experiences and desires’.^31^ As such, much like the early twentieth-century Anglo-American feminist press (which Lucy Delap and Maria Dicenzo have argued was not just made up of overlapping readerships but instead constituted a ‘single reading community that transcended national boundaries’), the rich print culture of late nineteenth-century free love worked to draw sex radicals from both Britain and America together to form a single, interconnected readership.^32^ Far from being incoherent or isolated, then, print gave diverse and distant free lovers sufficient opportunities to coalesce and cohere – to draw previously distinct British and American free love groups into a loose-knit, but cohesive Anglo-American free love community.

## 2. BUILDING A VOCABULARY

Within the context of pervasive debates about sex, marriage, and the family occurring in both America and Britain in the late nineteenth century, free love represented one of the most radical responses to questions about the state of marriage, and what constituted acceptable sexual behaviour.^33^ Unlike other contemporary reformers operating on both sides of the Atlantic who looked to improve existing systems of courtship and marriage, Anglo-American free lovers were hugely critical of the marriage institution as a whole. Rather than seeing marriage as a vital institution central to the entire social fabric, they claimed instead that it amounted to an unnatural and invasive form of oppression, through which mankind’s important ‘affinities and longings’ towards sexual companionship were ‘suspiciously watched, circumscribed, confined’.^34^ Agreeing that contemporary modes of sexual regulation were unnatural and injurious, they stressed in their writings that marriage was responsible for both individual suffering and a host of prominent social ills plaguing contemporary Britain and America, including prostitution, the prevalence of loveless and mercenary relationships, gender inequality, and racial degeneration.^35^ Their writings therefore called for orthodox sanctioned marriage to be completely overhauled, or done away with altogether – encouraging people to indulge in ‘normal, healthy, sexual gratifications’ at will, and to pursue romantic and intimate relationships completely free of interference from church and state.^36^ While their critics railed against what they saw as a campaign for ‘free lust,’ the free lovers themselves maintained that allowing people to participate in their intimate endeavours unchecked would provide a powerful ‘antidote to the deathly poison’ of married bondage, serving not only to make people happier and healthier, but also to motivate a host of positive changes that would improve society as a whole.^37^

But as historians have noted, ‘free love’ was a concept with diffuse and contested meanings in this period, as free lovers themselves struggled to negotiate a singular understanding of what it might mean to be sexually ‘free’.^38^ Reflecting the ‘conflicts, disputes, personality clashes, and ideological divisions’ John T. Cumbler has asserted were typical of contemporary reform communities, competing and often contradictory ideas about what free love looked like, and how it might best be lived day to day circulated between and within British and American free love circles.^39^ A number of key ideological fault lines thus emerged in Anglo-American free love thought, though not usually along national lines: monogamists in both Britain and America, for instance, maintained support for a system of lifelong, monogamous, but unsanctioned unions, clashing with varietist free lovers who argued that mankind’s complex sexuality could only be satisfied by a variety of sexual partners. Free lovers who emphasized the spiritual nature of intimate connections were at odds with other advocates who looked to define sexual and romantic activity in purely biological terms. Debates about the rights of women, though central to many free love debates, also proved divisive: feminist free lovers often campaigned for women’s sexual freedom in the same publications that hosted vocal opposition by other free lovers who looked to privilege male sexual expression.^40^ Indeed, Wendy Hayden has argued that many historians have resisted treating free love like other contemporary social movements precisely due to the fact that it contained such a diverse range of advocates, voicing such a wide range of seemingly inconsistent or contradictory views.^41^ ‘Free love’, then, was not a simple ask for free lovers. Articles in both British and American free love periodicals admitted that while they all agreed that orthodox marriage was fundamentally injurious, they were ‘not united at all . . . not unanimous at all, as to what institution should take its place’, and instead often occupied ‘diametrically opposite positions’ in their debates about what constituted the most desirable form of sex radical living.^42^ Given their own unease about the inconsistency of their views, it would certainly be easy to fall into the trap of seeing late nineteenth-century free lovers as an irrevocably incoherent and disjointed group – cleaved apart by both geographical distance and the inability to agree on a single idea of what free love might look like. But rather than surrendering to their differences, print gave free lovers space to manage such internal dissension and to negotiate and compromise in order to find common ground. In doing so, print became central to their efforts to create unity and to broadly coalesce as a transnational reform community. An exploration of their efforts therefore offers a valuable case study of the way that print could facilitate the building of a transatlantic reform community from even the most seemingly disparate and divided of groups.

Lacking opportunities for sustained, face-to-face discussion and debate, attempts to build a shared vocabulary for discussing free love focused on finding a way to gloss over the contradictions in their ideas, blunt their contextual differences, and discuss radical sexual reforms that suited the motivations and agendas of a diverse range of people. They sought to establish what common ground *did* exist between them, and to identify key points and guiding principles around which they could cohere. Among the *Lucifer* and *Adult* circles, the concept of sexual ‘freedom’ was one of the most important of these key ideas, with broad discussions of ‘freedom’, ‘liberty’, and ‘emancipation’ becoming central to much of their shared work. For many free lovers, the opposite of this ‘freedom’ and ‘emancipation’ was often ‘bondage’ and ‘slavery’, as radical authors on both sides of the Atlantic often asserted that mankind has been ‘enslaved’ by the controls and restraints placed upon sexuality by social and legal customs.

Of course, these shared terms were loaded with divergent meanings shaped by very different national contexts, and by the reform traditions tied to these contexts. American free lovers’ invocation of motifs of slavery and freedom, for example, were clearly much more influenced by close ties to American abolitionism and outspoken criticism of the Atlantic slave trade. As Eric Foner has shown through his interrogations of meanings of ‘freedom’ in nineteenth-century America, the country’s particular history of slavery led to these ideas being ‘employed by social movements of all descriptions as a master metaphor for inequality’ when discussing issues of personal liberty and the rights of American citizens in this period.^43^ Indeed, Moses Harman claimed that his campaigns for free love could be seen as ‘The New Abolition Movement’, and drew clear links between attempts to abolish chattel slavery and calls for sexual reform.^44^ Harman was certainly not alone, and scholars have outlined the particular influence of Garrisonian abolitionism on feminist free love agitation in the period, stating that it helped shape women’s awareness of the similarities between chattel slavery and the bonded conditions of wives in contemporary American society.^45^

Like their American counterparts, *Adult* contributors spoke of their campaigns for sex reform in the same terms of bondage and emancipation – an early journal subtitle announced it was formed as a ‘Crusade Against Sex-Enslavement’, while authors argued that free love would emancipate married people from ‘the original, purely slavish bond’.^46^ As Richard Huzzey has shown, the use of this kind of rhetoric (common to many social movements in Britain during this period) had links to Britain’s identity as an ‘anti-slavery state’, and the abolition of the Atlantic slave trade.^47^ But these rhetorical choices were further shaped by the geopolitical context of an expansive and expanding British empire, which facilitated free lovers’ use of a vocabulary Antoinette Burton argues would have been ‘steeped in racial metaphors and civilising tropes’.^48^ Discussions taking place in British free love publications about the ‘slavery’ of married women, in particular, were imbued with broader concerns about the white slave trade associated with prostitution, and parallels between British women and the Eastern Harem that had first been drawn over a century earlier by Mary Wollstonecraft in her ‘Vindication of the Rights of Women’ (1792).^49^

But print allowed them to negotiate such differences: free lovers used their journals as a space in which to construct a version of freedom that was not specific or well defined and was not tied to a particular vision of what being sexually ‘free’ might mean in practical terms. Instead, it was often something rather more nebulous – blunting both geographical difference and ideological inconsistency, authors and correspondents worked with a vague and malleable conceptualization of freedom that acted as a core around which even those free lovers engaging in the most contentious debates could unite. Reflecting this, for example, the *Adult*’s editor George Bedborough would only go as far as to speak of freedom as ‘the absence of external restraint and compulsion’ in matters of sex, emphasizing the importance of ‘individual sovereignty in individual concerns’ rather than advocating for a specific free love lifestyle.^50^ This corresponded with other *Adult* writers who were similarly unwilling to define freedom in solid terms; Frederick Rockell objected to a single definition of freedom being entered into the society’s constitution as one person ‘may have one idea, and others may have different ideas’.^51^*Lucifer*’s editor Moses Harman was often similarly oblique, and he asserted that the work of the free lover was simply to ensure people had ‘liberty’ (whatever that might mean) from the ‘slavery’ of marriage and restrictive sexual mores.^52^ Print therefore not only allowed heterogeneous voices opportunities to debate different ideas about freedom, but also worked as a neutral space through which they could compromise on a shared version of freedom that would, in the words of one author, ‘convey a meaning to all – not necessarily the same meaning, but some meaning to each of us’.^53^

Such a flexible understanding of freedom was a crucial tool in Anglo-American free love discourse. It spoke closely to their shared belief in the oppressive nature of orthodox marriage, and the potential dangers of limiting the expression of what they saw as mankind’s vital, natural sexuality. But, perhaps even more importantly, by being so imprecise it was also broad enough to allow them to gloss over their internal points of contention about the right way to be sexually free. It therefore allowed far-flung free lovers to offset the conflict and inconsistency of their contradictory ideas about appropriate free love lives, and acted as an important, unifying principle for a group of sex radicals wrestling with diverse agendas and motivations. As British free lovers explicitly stated, this transatlantic community was held together by their willingness to embrace ‘every lover of freedom, whatever ideal he may cherish’.^54^

While it may not have provided free lovers with specific details on how to structure their own relationships, this loose version of ‘freedom’, negotiated through the pages of their journals, was nevertheless hugely important to the construction of one the key foundations of this reform community – a collective identity. Ambiguous enough to function as a common goal shared by a diverse range of reformers on both sides of the Atlantic, the idea that they were united in the fight for sexual freedom helped to foster a sense of affinity, of sameness, that brought together free lovers from both Britain and America through the pages of *The Adult* and *Lucifer*.^55^ American free lovers, for example, wrote clearly of their feelings of friendship and comradeship for their English ‘cousins’ and ‘co-workers’ in the Legitimation League. Emphasizing their shared commitment to ‘liberty’ from the confines of marriage, Moses Harman spoke of feelings of ‘friendship, of comradeship, of love’ for his British allies in what he saw as ‘the holiest of Holy Wars, the war against . . . the mother of all slavery – sex-slavery’.^56^ Diminishing distinctions between British and American free love circles, *Lucifer* editorials asserted that Anglo-American free love was not ‘a mere British-American alliance’, but, regardless of ‘nationality, sex, or religious, political, or philosophical opinion’ should instead be understood as an ‘alliance of the friends of liberty’.^57^ British free lovers similarly saw themselves as part of a united, Anglo-American front. *Adult* editorials frequently highlighted their feelings of solidarity and friendship with their American peers, and were explicit about their desire to act as ‘splendid warriors of freedom’ in the same vein as prominent American sex radicals such as Moses Harman, Ezra Heywood, and Lois Waisbrooker.^58^ Therefore, rather than seeing themselves only as distinct entities, confined to operating within a specific national context, print allowed these reformers opportunities to emphasize their shared endeavours and common goals. Anglo-American free lovers, then, found a way to coalesce behind a common cause through the pages of their journals, and came to define themselves as ‘transatlantic comrades’ in the same battle for sexual freedom.^59^

## 3. COMMON ENEMIES, AND THE CONSTRUCTON OF A COLLECTIVE IDENTITY

However, the competing conceptions of what exactly it meant to be sexually ‘free’ were not the only divisions within the community that needed to be worked through. These free lovers also had to find ways to overcome the fact that their specific ideas about what they were fighting *against* were also often complex and contradictory. It would be easy to assume that these divisions were connected to national context, linked to the differing social, legal, and religious contexts of contemporary Britain and America; reflecting, perhaps, the higher rates of divorce Michael Grossberg has charted in the United States compared to Britain at this time, or the differing religious cultures of Britain, with its Church of England, and America, where the constitution had ratified the separation of church and state.^60^ In fact, there was little difference along these lines, and instead they tended to revolve around the different personal motivations and agendas that cut across national boundaries. For some free lovers, for example, it was the permanence of the marriage tie that rendered it oppressive and tyrannous. *Adult* correspondents criticized ‘perpetual marriage’ and the idea that ‘a man and woman’s love for one another must be given once and for all, and exclusively’, mirroring American radicals’ discussions of how ‘all the changing conditions and epochs of a long life’ rendered it nearly impossible to maintain exclusive, lifelong desire for a single person of the opposite sex.^61^ Others were suspicious of what they saw as the insidious legal and ecclesiastical forces responsible for policing sexual expression; British free lover Leighton Pagan spoke of marriage’s role in imposing injurious ‘legal bonds and sordid pressures’ onto an individual’s romantic life, while Moses Harman argued that marriage was ‘the last refuge and fortress, or stronghold, in, by and through which Ecclesiasticism hopes to perpetuate its power over mankind’, perpetuating too the power of the ‘despotic and invasive state’.^62^ Many more were concerned with the inequality and subjugation faced by women, in particular, within the existing marriage system. Resonating with the wider feminist criticism of the unequal and oppressive conditions of contemporary matrimony occurring in this period, many in this community spoke out in their articles and letters to criticize the institution of marriage for depriving middle-class women of their right to sexual self-determination and bodily autonomy, and the freedom to express their own natural sexuality.^63^

But, again, free lovers used their publications as a space in which they could work to define a set of common adversaries against which diverse Anglo-American sex radicals could present a united front – what Judy Greenway calls the ‘dystopian other, unfree love’, which they believed was responsible for causing such personal and social harm.^64^ In order to sidestep the multiplicity of their individual views, many of the free lovers’ shared challenges and criticisms were aimed at big, faceless, conceptual ‘enemies’; broad, inclusive, contextually non-specific targets which would allow free lovers of all persuasions to feel as though they were part of a co-ordinated and coherent reform effort. Both British and American radicals, for example, railed against somewhat abstract notions of ‘conventionalism’ and ‘respectability’ as forces of sexual control in their work. Anonymous British free lover ‘Sagittarius’, for instance, spoke out against the culture of ‘self-suppression amidst conventionalism’,^65^ arguing that mankind’s vital sexuality had been ‘chained to earth by myriad bonds of . . . convention, ‘respectability’, and ignorance’.^66^ These attacks closely mirrored misgivings voiced by prominent American free lovers in writings published on both sides of the Atlantic. E. C. Walker described the stifling nature of sanctioned monogamous marriage as representing a form of ‘abject slavery to the gods of mock propriety’,^67^ while Canadian R. B. Kerr criticized those who ‘yield obediency to . . . the conventionalism of artificial human society’, and outlined the role of ‘respectability’ in perpetuating the power of the injurious ‘social machine’.^68^ Circulating such a shared and sympathetic set of criticisms through their publications, levelled at such an indistinct and ambiguous target, performed an important unifying function for Anglo-American free lovers, allowing often disparate and geographically diverse reformers the chance to assert that they were fighting the same fight against the tyranny of convention and taboo, and to define themselves as part of a bigger challenge to existing sexual customs.

Other antagonists, though, provided the community with a more specific target around which they could focus their shared endeavours. In particular, free lovers working on both sides of the Atlantic united to protest against the activities of notorious American vice reformer Anthony Comstock. For free love advocates working in the USA, Comstock and his campaigns to supress vice through censorship were a real, pressing, and long-held concern. From the 1880s he had spearheaded the crusade against outspoken free lovers like Moses Harman and Ezra Heywood, both of whom had been imprisoned and sentenced to periods of hard labour for publishing and distributing materials discussing sex.^69^ Indeed, he pursued spiritualist free lover Ida Craddock with such ‘vindictive zeal’^70^ that she committed suicide in 1902 to avoid being returned to prison on obscenity charges for a second time, blaming Comstock by name in a public note which likened his crusade against free love to ‘a medieval Inquisition’.^71^ Despite the fact that British authorities were increasingly concerned with the traffic in obscene materials at this time (Collette Colligan has outlined the proliferation of legislation designed to impede the circulation of obscene texts and images)^72^ the *Adult* circle was generally less troubled by the censors. Though, as Elizabeth Miller has shown, they were at the vanguard of debates about free speech and a free press, they only suffered one arrest – that of George Bedborough on obscenity charges in 1898.^73^ Unlike the protracted and intense persecution faced by their American counterparts, they only had one run-in with Comstock during their interventions into free love debates in America, when a small shipment of 500 copies of a pamphlet by Legitimation League founder Oswald Dawson were seized by US customs under the Comstock Act.

However, despite the difference between the publishing contexts of Britain and America, British radicals also sought to depict themselves as direct enemies of Comstock in their publications, engaged in the same tumultuous battle against censorship and the suppression of open discussion of sex being fought by their American comrades. They launched blistering responses to news of Comstock’s activities, attacking him in editorials and articles for creating laws ‘exploited by every bigot’ in order to ‘kill frankness and truth’ in discussions of sex.^74^ Furthermore, to further consolidate the idea that they were part of a shared fight against Comstock, George Bedborough blamed him directly for his arrest, stating that obscenity charges had been brought against him due to the fact that Comstock had reached over the ocean to persecute him with ‘longer arms than [he] fancied’ – despite the fact that, in reality, Comstock had absolutely nothing to do with his prosecution.^75^ Comstock therefore came to be almost mythologized in the transatlantic free love publications as a ceaseless and brutal foil of freedom, acting as a touchstone for Anglo-American free lovers looking for a common enemy against which to unite. Regardless of the realities of their relationship with him, or the real level of threat he posed, Comstock functioned as an adversary, a *bête noire*, against which diverse free lovers with different motivations and concerns could present a united and coherent front.

Creating such an inclusive set of common adversaries and abiding to such broad notions of ‘freedom’ and ‘bondage’ in their work offered free lovers in both America and Britain the opportunity to strengthen and consolidate the ties between them, to emphasize common ground, and to foster a tangible sense of affinity, kinship, and shared endeavour. In short, their rhetorical imprecision and willingness to compromise throughout their publications allowed them to assert that, regardless of their complex individual beliefs about how free love should be lived, their competing motivations and priorities, and the different national contexts in which they were working, sex radical reformers on both sides of the Atlantic were engaged in the same campaign, for the same cause. While existing scholarship on both British and American free love campaigns has often asserted that they should be characterized by incoherence or isolation, a brief interrogation of the ideological relationship forged through connections the *Lucifer* circle and the *Adult* shows that even without opportunities for face to face discussion these free lovers actively pursued the establishment of an important collective identity through print. Through print, then, we can chart the specific processes of negotiation and compromise that allowed diverse free lovers to surmount both national boundaries and ideological inconsistency in order to form a truly transatlantic reform community.

## 4. CONCLUSION

Ultimately, this transatlantic relationship – so optimistically forged to provide ‘coherence, courage, and hope’ to reformers in both Britain and America – was not enough to save *The Adult*: following Bedborough’s conviction, it collapsed in 1900. *Lucifer* (already one of the only remaining dedicated free love periodicals in America by the end of the century) carried on until 1907, eventually shifting its focus away from sexual reform before being reborn as *The American Journal of Eugenics*. While the endeavours of free lovers may have reached a rather unremarkable end, analysis of their journals can nevertheless provide important new insights into the relationships forged on either side of the Atlantic – as this article has shown, for a time they were able to build a lively and cohesive community of Anglo-American free lovers who were committed to radical sexual reform. Through print alone they forged a common identity, a shared vocabulary, and a broad but cohesive version of free love to work with; a process that my research has shown was even more important to a community that was not only financially precarious, but socially controversial, and fraught with potential divisions and contradictions. One of the key contributions of this work, then, is a methodological challenge. Historians have often accessed histories of free love through restrictive accounts of personal experiences, or have limited accounts of free love to the fringes of studies of broader reform movements; an approach which has often led to free love being depicted in historical work as irrevocably fragmented, isolated, and disparate. An interrogation of the ways in which their rich periodical network drew together reformers from Britain and America, though, asserts that studying print can give us new and important stories to tell about fin-de-siècle free love that mark it out as a cohesive transatlantic reform movement with an identity and ideology of its own. But the impact of these findings goes further than just calling for a new approach to the specific history of free love. Instead, by charting the role of print in transforming scattered free lovers into a radical reform community, it encourages historians to look again at other contemporary social reform communities often considered too small, too fringe, or too incoherent to truly be part of international reform networks. Studying such communities through print, I argue, will allow us to move away from narratives of isolation, conflict, and marginality, and instead chart threads of unity and cooperation across borders.

## Footnotes

1 ‘Indictments Against Bedborough’, *Lucifer, the Light-Bearer*, 17 September 1898, pp. 300–301.

2 Moses Harman, ‘Help Fight Freedom’s Battle’, *Lucifer, the Light-Bearer*, 9 July 1898, p. 210.

3 While some of these sex radicals avoided the terminology of ‘free love’ to describe their work due to its connotations of promiscuity, many of those campaigning for radical changes to marriage embraced the title of ‘free lover’; a title which summed up their ideas about the injustices of contemporary marriage and courtship, and the importance of living a sexual life free from external constraints. For a discussion of terminology and the use of ‘free lover’ and ‘sex radical’ see Joanne E. Passet, *Sex Radicals and the Quest for Women’s Equality* (Urbana and Chicago, IL: University of Illinois Press, 2003), p. 2.

4 ‘Some Letters, a Constitution, and a Speech’, *The Adult*, January 1898, p. 136.

5 Lucy Delap, *The Feminist Avant-Garde: Transatlantic Encounters of the Early Twentieth Century* (Cambridge: Cambridge University Press, 2007), p. 55.

6 Delap, *The Feminist Avant-Garde*; John Fagg, Matthew Peter, and Robin Vandome, ‘Networks and the Nineteenth-Century Periodical’, *American Periodicals*, 23 (2013), 93–104.

7 See, for example, Sandra Stanley Holton, ‘Free Love and Victorian Feminism: The Divers Matrimonials of Elizabeth Wolstenholme and Ben Elmy’, *Victorian Studies*, 37 (1994), 199–222; Karen Hunt, *Equivocal Feminists: The SDF and the Woman Question 1884–1911* (Cambridge: Cambridge University Press, 1996); Jane Lewis, ‘Intimate Relations Between Men and Women: the Case of H. G. Wells and Amber Pember Reeves’, *History Workshop Journal*, 37 (March 1994), 76–98; Christine Collette, ‘Socialism and Scandal: the Sexual Politics of the Early Labour Movement’, *History Workshop Journal*, 23 (Spring 1987), 102–11.

8 Ginger S. Frost, *Living in Sin: Cohabiting as Husband and Wife in Nineteenth-Century England* (Manchester and New York, NY: Manchester University Press, 2008).

9 See Wendy Hayden, *Evolutionary Rhetoric: Sex, Science, and Free Love in Nineteenth-Century America* (Carbondale, IL: Southern Illinois University Press, 2013); Jesse Battan, ‘“Socialism Will Cure All But An Unhappy Marriage”: Free Love and the American Left, 1850–1910’, in *Meetings and Alcôves: The Left and Sexuality in Europe and the United States since 1850*, ed. by Jesse Battan, Thomas Bouchet, and Tania Régin (Dijon: Editions Universitaires de Dijon, 2004), pp. 29–46.

10 Leslie Butler, *Critical Americans: Victorian Intellectuals and Transatlantic Liberal Reform* (Chapel Hill, NC: University of North Carolina Press, 2009); Daniel Rodgers, *Atlantic Crossings: Social Politics in a Progressive Age* (Cambridge, MA: Belknap Press of Harvard University Press, 1998).

11 Margaret Beetham, ‘Periodicals and the New Media: Women and Imagined Communities’, *Women’s Studies International Forum*, 29 (2006), 231–40 (p. 232).

12 See, for example, Sarah L. Silkey, *Black Woman Reformer: Ida B. Wells, Lynching, and Transatlantic Activism* (Athens, GA: University of Georgia Press, 2015).

13 Delap, *The Feminist Avant-Garde*.

14 Elizabeth Carolyn Miller, *Slow Print: Literary Radicalism and Late Victorian Print Culture* (Stanford, CA: Stanford University Press, 2013), pp. 2–3.

15 For the history of *Lucifer* see Hal Sears, *The Sex Radicals*: *Free Love in High Victorian America* (Lawrence, KS: Regents Press of Kansas), pp. 53–64. For an overview of the founding of *The Adult* see Anne Humpherys, ‘The Journals That Did: Writing about Sex in the late 1890s’, *Interdisciplinary Studies in the Long Nineteenth Century*, 3 (2006) <https://doi.org/10.16995/ntn.450> [accessed 3 October 2018]

16 Laurel Brake and Julie F. Codell, ‘Introduction’, in *Encounters in the Victorian Press: Editor, Authors, Readers*, ed. by Laurel Brake and Julie F. Codell (Basingstoke: Palgrave Macmillan, 2004), pp. 1–7 (p. 5).

17 Ann Ardis and Patrick Collier, ‘Introduction’, in *Transatlantic Print Culture, 1880–1940: Emerging Media, Emerging Modernisms*, ed. by Ann Ardis and Patrick Collier (Basingstoke: Palgrave Macmillan, 2008), pp. 1–12.

18 Amanda Claybaugh, *The Novel of Purpose: Literature and Social Reform in the Anglo-American World* (Ithaca, NY: Cornell University Press, 2007), p. 2.

19 Janice Ruth Wood, *The Struggle for Free Speech in the United States, 1872–1915: Edward Bliss Foote, Edward Bond Foote, and Anti-Comstock Operations* (Oxford and New York, NY: Routledge, 2011), p. 103. The bankruptcy proceedings of Oswald Dawson, the founder of the Legitimation League, were covered by a number of newspapers. See, for example, ‘The Drowning Season’, *Lloyd’s Weekly Newspaper*, 17 September 1899.

20 Passet, *Sex Radicals*, p. 50.

21 Joanne E. Passet, ‘Power Through Print: Lois Waisbrooker and Grassroots Feminism’, in *Women in Print: Essays on the Print Culture of American Women from the Nineteenth and Twentieth Centuries*, ed. by James Philip Danky and Wayne A. Wiegand (Madison, WI: University of Wisconsin Press, 2006), pp. 229–50.

22 Bob Nicholson, ‘Transatlantic Connections’, in *The Routledge Handbook to Nineteenth-Century Periodicals and Newspapers*, ed. by Andrew King, Alexis Easley, and John Morton (London and New York: Routledge, 2016), pp. 163–74 (pp. 164–65).

23 See, for example, Lillian Harman, ‘Eve and her Eden’, *The Adult*, March 1898, pp. 32–35; Dora F. Kerr, ‘The Conversion of Mrs Grundy’, *The Adult*, May 1898, pp. 96–101; Tennessee Claflin, ‘Malthus’, *The Adult*, February 1898, pp. 9–12.

24 Moses Harman, ‘Editorial’, *Lucifer, the Light-Bearer*, 28 May 1898, p. 164.

25 William Duff, Letter in ‘Various Voices’, *Lucifer*, *the Light-Bearer*, 25 August 1897, p. 271.

26 John Badcock Jr., ‘Personal Responsibility’, *Lucifer, the Light-Bearer*, 5 January 1898, pp. 418–19.

27 William Platt, ‘Love and Business Principles’, *The Adult*, May 1898, pp. 112–13.

28 Lillian Harman, ‘The Legitimation League’, *Lucifer, the Light-Bearer*, 28 April 1897, p. 130.

29 Brake and Codell, ‘Introduction’, p. 2.

30 Jesse Battan, ‘“You Cannot Fix the Scarlet Letter on My Breast!”: Women Reading, Writing, and Reshaping the Sexual Culture of Victorian America’, *Journal of Social History*, 37 (2004), 601–24 (p. 607).

31 Battan, ‘You Cannot Fix the Scarlet Letter’, p. 614.

32 Lucy Delap and Maria DiCenzo, ‘Transatlantic Print Culture: The Anglo-American Feminist Press and Emerging “Modernities”’, in *Transatlantic Print Culture*, ed. by Ardis and Collier, pp. 48–65 (p. 56).

33 For information about contemporary debates about marriage in America see Claire Virginia Eby, *Until Choice Do Us Part: Marriage Reform in the Progressive Era* (Chicago, IL: University of Chicago Press, 2014); for the British context, see Lucy Bland, *Banishing the Beast: Feminism, Sex and Morality* (London: Tauris Parke, 2001), chapter 4.

34 Sagittarius, ‘“The Liars”: Or, Marriage at the Criterion Theatre’, *The Adult*, December 1897, p. 84.

35 For information on theories of racial degeneration and free love see George Robb, ‘The Way of All Flesh: Degeneration, Eugenics, and the Gospel of Free Love’, *Journal of the History of Sexuality*, 6 (1996), 589–603; Sarah Jones, ‘Gender, Reproduction, and the Fight for Free Love in the Nineteenth-Century Periodical Press’, in *Women in Magazines: Research, Representation, Production and Consumption*, ed. by Rachel Ritchie, Sue Hawkins, Nicola Phillips, and S. Jay Kleinberg (New York, NY and Oxford: Routledge, 2016), pp. 55–65.

36 Orford Northcote, ‘Music, Religion and Sex – II,’ *The Adult*, March 1898, p. 44.

37 E. C. Walker, ‘Love – Its Attractions and Expression’, *Lucifer, the Light-Bearer*, 21 April 1897, p. 121.

38 Passet, *Sex Radicals*, p. 2.

39 John T. Cumbler, *From Abolition to Rights for All: The Making of a Reform Community in the Nineteenth Century* (Philadelphia, PA: University of Pennsylvania Press, 2013), p. 8. Heather A. Haveman has also highlighted similar divisions in such nineteenth-century movements as those for abolition, temperance, pacifism, and prison reform. See Haveman, *Magazines and the Making of America: Modernization, Community, and Print Culture, 1741–1860* (Princeton, NJ: Princeton University Press, 2015), p. 201.

40 For a discussion of tensions around women and their capacity for sexual freedom in the British context see Jones, ‘Gender, Reproduction, and the Fight for Free Love’; for the USA see Passet, *Sex Radicals*.

41 Hayden, *Evolutionary Rhetoric*, p. 12.

42 Oswald Dawson, ‘Two Editorials’, *The Adult*, January 1898, p. 110; E. C. Walker, ‘Home Life, Health and Happiness’, *Lucifer, the Light-Bearer*, 20 January 1897, p. 21.

43 Eric Foner, ‘The Meaning of Freedom in the Age of Emancipation’, *The Journal of American History*, 81 (1994), 435–60 (p. 438).

44 Moses Harman, ‘The New Abolition Movement’, *Lucifer, the Light-Bearer*, 11 August 1900, pp. 245–46.

45 Passet, *Sex Radicals*, p. 65. For information on William Lloyd Garrison and his abolitionist politics see Henry Mayer, *All on Fire: William Lloyd Garrison and the Abolition of Slavery* (New York, NY: St Martin’s Press, 1998).

46
*The Adult*, September, 1897; Dora Kerr, ‘The Custom of Marriage’, *The Adult*, March 1899, p. 62.

47 Richard Huzzey, *Freedom Burning: Anti-Slavery and Empire in Victorian Britain* (Ithaca, NY: Cornell University Press, 2012), p. 81.

48 Antoinette Burton, *Burdens of History: British Feminists, Indian Women, and Imperial Culture, 1865–1915* (London: University of North Carolina Press, 1994), p. 2.

49 For an in-depth look at Wollstonecraft’s treatment of the issue of slavery see Moira Ferguson, *Colonialism and Gender Relations from Mary Wollstonecraft to Jamaica Kincaid: East Caribbean Connections* (New York, NY: Columbia University Press, 1993).

50 George Bedborough, ‘The Legitimation League’, *The Adult*, May 1898, p. 119.

51 D. Oliver, ‘The Annual Meeting and Dinner’, *The Adult*, January 1898, p. 112.

52 Moses Harman, ‘A Free Man’s Creed’, *Lucifer, the Light-Bearer*, 7 April 1897, p. 106. Harman’s commitment to anti-slavery appears to be directly linked to his own long-held abolitionist views. Harman was run out of Crawford County, Ohio, in the early 1860s for his unpopular and outspoken advocacy of anti-slavery campaigns. For information on Harman’s role in abolitionist movements see Sears, *The Sex Radicals*, p. 30.

53 Oliver, ‘The Annual Meeting’, p. 119.

54 Bedborough, ‘The Legitimation League’, p. 119.

55 For a discussion of the importance of collective identities to social movements, see Francesca Polletta and James M. Jasper, ‘Collective Identity and Social Movements’, *Annual Review of Sociology*, 27 (2001), 283–305.

56 ‘Legitimation League’s Annual Meeting’, *Lucifer, the Light-Bearer*, 21 May 1898, p. 153.

57 ‘George Bedborough’s Call for Help’, *Lucifer, the Light-Bearer*, 16 July 1898, p. 228.

58 Bedborough, ‘The Legitimation League’, p. 119.

59 Oswald Dawson, *Personal Rights and Sexual Wrongs* (London: Wm. Reeves, 1897), p. 31.

60 Michael Grossberg, *Governing the Hearth: Law and the Family in Nineteenth-Century America* (Chapel Hill, NC: University of North Carolina Press, 1988), p. 251; Steven Green, *The Second Disestablishment: Church and State in Nineteenth-Century America* (Oxford and New York, NY: Oxford University Press, 2010), p. 11.

61 George Bedborough, ‘What the Legitimation League Means’, *The Adult*, June 1897, p. 3. Edwin C. Walker, ‘The “Monogamic Ideal” and the “Ideal Man”’, *The Adult*, June 1898, p. 140.

62 Leighton Pagan, ‘To the “Obscure Judes” and Distracted “Sues”’, *The Adult*, June 1897, p. 6; Moses Harman, ‘Why I Oppose Marriage’, *The Adult*, January 1898, p. 171.

63 Bland, *Banishing the Beast*, p. 124.

64 Judy Greenway, ‘Speaking Desire: Anarchism and Free Love as Utopian Performance in Fin de Siècle Britain’, in *Anarchism and Utopianism*, ed. by Laurence Davis and Ruth Kinna (Manchester: Manchester University Press, 2009), pp. 153–70 (p. 154).

65 Sagittarius, ‘Sexual Freedom in Relation to Women and Economics’, *The Adult*, September 1897, p. 28.

66 Sagittarius, ‘The Liars’, p. 84.

67 E. C. Walker, *What the Young Need to Know: A Primer of Sexual Rationalism* (Chicago, IL: M. Harman, 1897), p. 4.

68 Moses Harman, ‘Respectability Again’, *Lucifer, the Light-Bearer*, 6 January 1900, p. 412.

69 John D’Emilio and Estelle B. Freedman, *Intimate Matters: A History of Sexuality in America* (Chicago, IL: University of Chicago Press, 1997), p. 164.

70 Comstock’s campaign against Craddock is outlined in detail in Vere Chappell, *Sexual Outlaw, Erotic Mystic: The Essential Ida Craddock* (San Francisco, CA: Weiser Books, 2010), p. 224.

71 Leigh Eric Schmidt, *Heaven’s Bride: The Unprintable Life of Ida C. Craddock, American Mystic, Scholar, Sexologist, Martyr, and Madwoman* (New York, NY: Basic Books, 2010), p. 220. The entire public letter was reprinted in *Lucifer, the Light-Bearer*, 13 November 1902, pp. 344–46.

72 Collette Colligan, *The Traffic in Obscenity from Byron to Beardsley: Sexuality and Exoticism in Nineteenth-Century Print Culture* (Basingstoke: Palgrave MacMillan, 2006), pp. 11–12.

73 Miller, *Slow Print*, pp. 257–98.

74 George Bedborough, ‘Comstock Rex’, *The Adult*, April 1898, p. 63.

75 ‘Comstockism at Work in England’, *Lucifer, the Light-Bearer*, 19 June 1898, p. 185.

